# A Case of Pneumocystis jirovecii Pneumonia Requiring Veno-Venous Extracorporeal Membrane Oxygenation (VV-ECMO) Management in a Young Patient With Aortic Inflammation Syndrome

**DOI:** 10.7759/cureus.93396

**Published:** 2025-09-28

**Authors:** Mamiko Kondo, Wakiko Aisaka, Hirofumi Terada, Hiroki Takahashi, Satoshi Kazuma

**Affiliations:** 1 Department of Intensive Care Medicine, Sapporo Medical University School of Medicine, Sapporo, JPN; 2 Department of Emergency Medicine, Sapporo Medical University School of Medicine, Sapporo, JPN; 3 Department of Anesthesiology, Kushiro City General Hospital, Kushiro, JPN; 4 Department of Rheumatology and Clinical Immunology, Sapporo Medical University School of Medicine, Sapporo, JPN

**Keywords:** aortic inflammation syndrome, immunosuppressive therapy, mechanical ventilation, pneumocystis jirovecii pneumonia, venovenous-extracorporeal membrane oxygenation

## Abstract

Pneumocystis jirovecii pneumonia (PCP) occurs in immunocompromised patients. It is classified into cases in human immunodeficiency virus (HIV)-positive (HIV-PCP) patients and HIV-negative (non-HIV-PCP) patients. Non-HIV-PCP is associated with particularly poor prognosis, and case reports using veno-venous extracorporeal membrane oxygenation (VV-ECMO) are scarce.

This case report describes a 25-year-old female patient with a history of taking multiple immunosuppressive medications who was diagnosed with PCP. She received antibiotic treatment, but her hypoxemia worsened, and she was admitted to the intensive care unit. Non-invasive respiratory therapy failed to improve hypoxemia, so endotracheal intubation and mechanical ventilation were initiated. However, adequate oxygenation was not achieved. Despite immunodeficiency, the patient was young and had no other organ failure. Therefore, the initiation of VV-ECMO was decided upon. After 20 days of VV-ECMO management, there were no major complications, and respiratory abnormalities improved. In cases of non-HIV-PCP presenting with life-threatening respiratory failure, VV-ECMO initiation should be considered.

## Introduction

Pneumocystis jirovecii pneumonia (PCP) is a disease with a high mortality rate that primarily affects immunocompromised patients. While it primarily occurs in human immunodeficiency virus (HIV)-positive (HIV-PCP) patients, due to the widespread use of immunosuppressive therapy, the incidence rate has also increased in HIV-negative (non-HIV-PCP) patients [1]. According to previous reports, the mortality rate is significantly higher in non-HIV-PCP patients than in HIV-PCP patients (27% vs. 4%) [2]. HIV-PCP patients tend to be younger, and their immunosuppression is reversible with antiretroviral therapy. In contrast, non-HIV-PCP patients often have underlying comorbidities, and their disease progression is more rapid than HIV-PCP, which is thought to be the cause of their higher mortality rate.

It is estimated that 30-40% of PCP patients require tracheal intubation due to respiratory failure [2]. Mechanical ventilation in patients with hematologic malignancies or immunodeficiency is generally known to worsen prognosis [3]. Therefore, critical conditions are avoided using non-invasive positive pressure ventilation (NPPV) or high-flow nasal cannula (HFNC) oxygen therapy. However, if hypoxemia is severe enough, these measures may be ineffective, and tracheal intubation becomes necessary. Given the extremely poor prognosis, extracorporeal membrane oxygenation (ECMO) is generally not indicated [4]. Nevertheless, in cases where the patient is young and has no sepsis due to bacterial infection or other organ failure, ECMO-based aggressive treatment should be considered for life-saving purposes.

In this report, we describe a case of severe PCP in a young patient with aortitis syndrome who had received long-term immunosuppressive therapy. The patient was successfully resuscitated with ECMO-assisted ventilation and returned to society.

## Case presentation

A 25-year-old woman with aortitis syndrome had been taking 20 mg of prednisolone daily, as well as several immunosuppressive drugs (mycophenolate mofetil 2000 mg/day, cyclosporine 100 mg/day, and tocilizumab 162 mg/day), for 10 years. She was admitted to the department of internal medicine at this hospital due to a suspicion of interstitial pneumonia, as evidenced by dyspnea on exertion for two days and predominant left lung ground glass opacity on a chest X-ray. Upon admission, the respiratory rate was 16 breaths per minute and the SpO₂ was 92% with a 3 L/min nasal cannula. PCP or cytomegalovirus (CMV) pneumonia were suspected, and treatment with prednisolone at 80 mg/day and sulfamethoxazole/trimethoprim (20 mg/kg/day in trimethoprim equivalent) and ganciclovir was initiated. The treatment response was poor, so the patient was transferred to the intensive care unit (ICU) on the fourth day of hospitalization. There, high-dose steroid therapy of 1000 mg/day for three days was initiated. On ICU admission, the patient was on HFNC therapy with a FIO₂ of 0.5, and the flow rate was 50 L/min. The respiratory rate was 36 breaths per minute and the SpO₂ was 86%. The heart rate was 103 beats per minute, the blood pressure was 125/65 mmHg, and the body temperature was 36.2°C. The consciousness was clear. Blood tests showed no signs of renal or coagulation failure. There were elevated levels of β-D-glucan (73.7 pg/mL), KL-6 (530 U/mL), SP-A (126 ng/mL), and SP-D (139 ng/mL) (Table 1).

**Table 1:** Findings at admission examination KL6: sialylated carbohydrate antigen KL-6; APTT: activated partial thromboplastin time; FDP: fibrinogen degradation product; BUN: blood urea nitrogen

Laboratory value	Data	Reference range
WBC (×10^3^/µL)	17.2	3.3~8.6
CRP (mg/dL)	<0.1	<0.14
Hb (g/dL)	12.7	11.6~14.8
Plt (×10^3^/µL)	197	158~348
T-bil (mg/dL)	0.9	0.4~1.5
LDH (U/L)	489	124~222
BUN (mg/dL)	26	8~20
Cr (mg/dL)	0.75	0.46~0.79
PT-INR	1.01	0.85~1.15
APTT (sec)	29.0	21.9~35.9
FDP (µg/ml)	<2.0	<5
β-D-glucan (pg/mL)	73.7	≦11
KL-6 (U/mL)	530	<500
SP-A (ng/mL)	126	<43.8
SP-D (ng/mL)	139	<110

**Figure 1:**
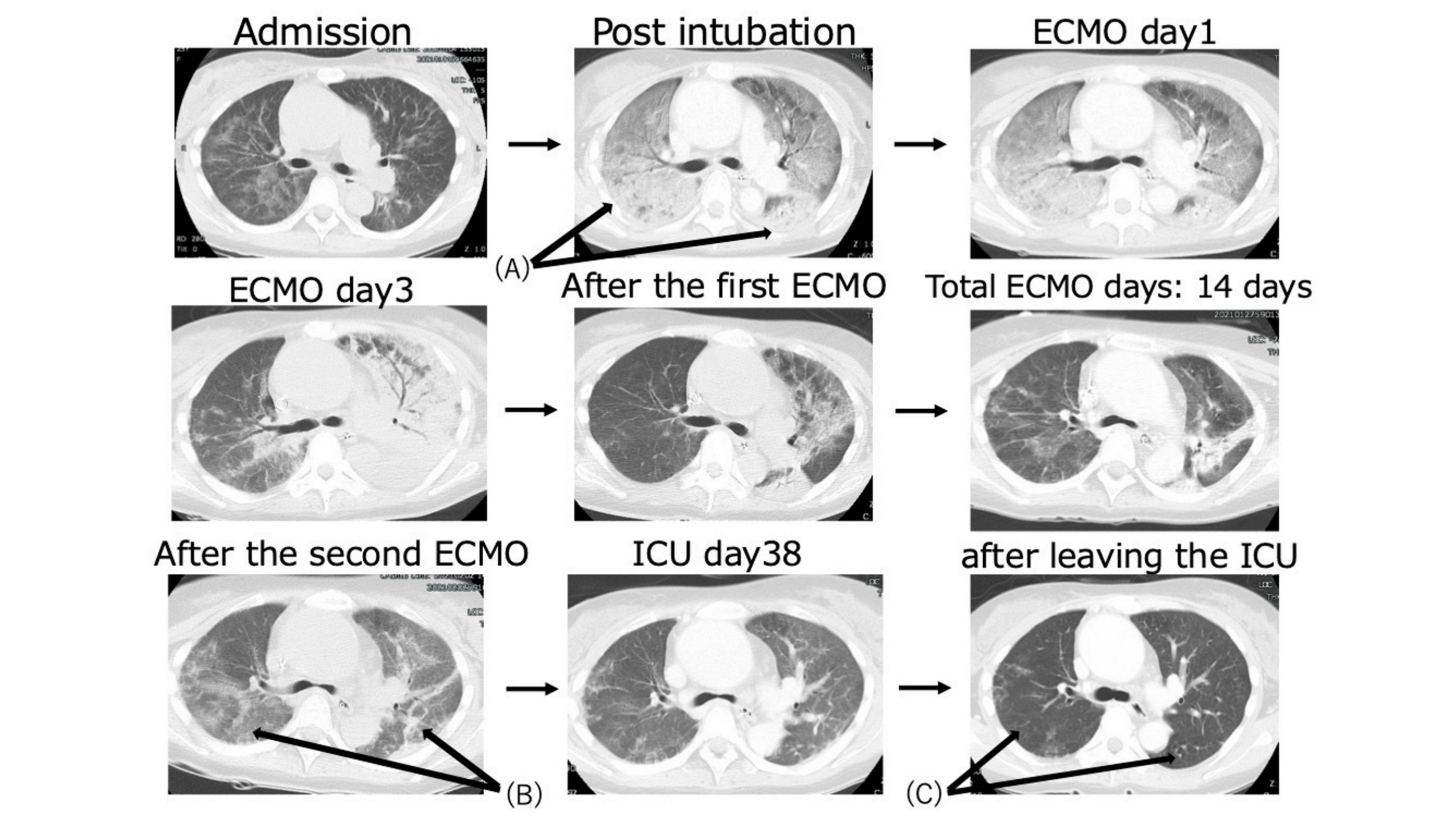
Changes in computed tomography findings At the time of intubation, the shadows in both lung fields had worsened (A). At the time of the second ECMO initiation, the consolidation in both lung fields had worsened again (B). By the time of discharge from the ICU, the ground-glass opacities in both lung fields had almost completely resolved (C). ECMO: Extracorporeal membrane oxygenation

CMV antigen was negative, so ganciclovir was discontinued. Before ICU admission, considering bacterial pneumonia, piperacillin/tazobactam and levofloxacin were administered, but all bacterial cultures were negative. Given the patient's immunodeficiency and respiratory failure, tracheal intubation was avoided as much as possible, and HFNC therapy and NPPV were continued. However, due to persistent hypoxemia, tracheal intubation and mechanical ventilation were initiated on the fifth ICU day, after informing the patient (Figure 1). Ventilator settings were adaptive support ventilation, tidal volume per predicted body weight 6mL/kg, and lung-chest compliance 34 mL/cmH₂O. Attempts were made to suppress harmful inspiratory efforts using deep sedation and muscle relaxants.

**Figure 2:**
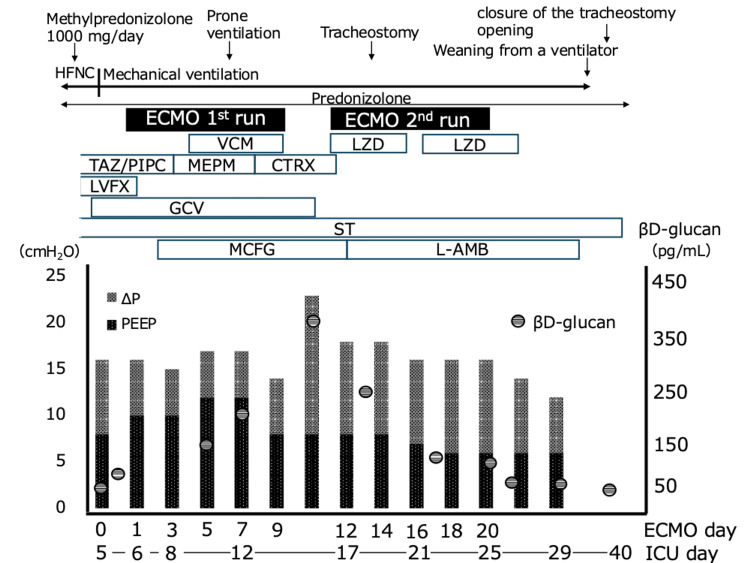
Timeline of the clinical course and treatments HFNC: high-flow nasal cannula, ECMO: extracorporeal membrane oxygenation, PEEP: Positive end expiratory pressure, TAZ/PIPC: tazobactam/piperacillin, MEPM: meropenem, CTRX: ceftriaxone, VCM: vancomycin, LZD: linezolid, LVFX: levofloxacin,  GCV: ganciclovir, ST: sulfamethoxazole- trimethoprim, MCFG: micafungin, L-AMB: liposomal amphotericin B

On the sixth ICU day, the FIO₂ was 0.7 and the PaO₂/FIO₂ ratio was 114 with a positive end-expiratory pressure (PEEP) of 10 cm H₂O. There were significant fluctuations in oxygenation due to changes in body position. Since there had been no improvement in respiratory status up to this point, ECMO was initiated (blood flow of 3.5 L/min) in anticipation of progressive hypoxemia and the lung lesions' long-term recovery. Ventilation was set to so-called "lung rest" conditions (Figure 2). PEEP was set by transpulmonary pressure measurement and supine ventilation. Empirical antimicrobial therapy was continued. After initiating VV-ECMO and transitioning to lung rest settings, the driving pressure decreased from over 20 cmH₂O to a target of 10-14 cmH₂O within 48 hours.

The patient was weaned from ECMO on the 13th ICU day. However, oxygenation was poorer than expected (PaO₂/FIO₂ ratio of 105, PEEP of 8 cm H₂O, and FIO₂ of 0.6, driving pressure > 20 cm H_2_O), and the patient continued to breathe with effort. Therefore, ECMO was reintroduced on the 16th ICU day. A tracheostomy was performed on the 18th ICU day. Against a background of persistent refractory pulmonary lesions and elevated β-D-glucan levels, alveolar lavage fluid testing was added. Sustained elevation of β-D-glucan levels led to suspicion of a zygomycosis complication, which is not covered by MCFG, prompting a change in antifungal therapy to amphotericin B. Administration of this drug commenced on the 18th day of ICU admission. Subsequently, β-D-glucan levels decreased from 452.7 pg/mL (peak level, on the 7th ICU day) to 35 pg/mL (on the 36th ICU day).

The patient subsequently showed clinical improvement and was weaned off ECMO on the 25th ICU day and off the ventilator on the 34th ICU day. On the 40th day in the ICU, the patient transitioned to nasal cannula oxygenation and was discharged the following day. During her ICU stay, microbiological examinations were consistently negative except for a positive PCR test for PCP. After 87 days of hospitalization, the patient was discharged and is now returning to society after continuing to receive outpatient care. She was able to return to work and daily life despite ongoing treatment.

## Discussion

This report emphasizes the importance of introducing VV-ECMO in cases of respiratory failure in non-HIV-associated PCP patients with severe immunodeficiency. The report also emphasizes the necessity of antimicrobial therapy for PCP and advanced intensive care.

A retrospective study of 240 cases of PCP diagnosed over a 17-year period reported the following [2]. The overall in-hospital mortality rate was 25.4%, the proportion of cases requiring ECMO was 4.6%, the mortality rate in cases requiring ventilator management was 60.5%, and the mortality rate in cases requiring ECMO was 81.8%. These findings suggest that the prognosis for PCP patients is generally poor.

Additionally, a 10-year retrospective study published in 2019 compared the outcomes of HIV-PCP patients and non-HIV-PCP patients who required ECMO [5]. The study reported that the survival rate among HIV patients who required ECMO was 50%, while the survival rate among non-HIV-PCP patients was 8%. The study concluded that HIV patients have a favorable prognosis and that ECMO should not be withheld. Among non-HIV PCP patients, one-third were successfully weaned off ECMO; however, there was a tendency toward higher frequencies of acute hemodialysis, and many patients experienced complications and continued to suffer from multi-organ failure even after being weaned off ECMO.

Non-HIV-PCP patients often have underlying conditions such as organ transplantation, malignancy, or autoimmune disease. Compared to HIV-PCP patients, non-HIV-PCP patients tend to have more rapid disease progression and frequently present with more severe hypoxemia and shock. The high mortality rate among non-HIV-PCP patients can be attributed to older age and the presence of multiple comorbidities compared to HIV patients [6].

In this case, the patient was a young steroid user with concomitant aortic inflammation syndrome who did not have life-threatening hypoxemia. Therefore, tracheal intubation was initially withheld, and HFNC therapy was continued for several days. This is an area for improvement in the future. After tracheal intubation, hypoxemia became apparent, leading to ECMO initiation. Generally, the Extracorporeal Life Support Organization (ELSO) guidelines indicate that the criteria for VV-ECMO initiation include a partial pressure of oxygen (P/F) ratio of 80 mmHg or lower, reversible pathology, and the absence of severe immunodeficiency [7]. In this case, the P/F ratio was 114 at the time of ECMO initiation; however, there were significant fluctuations in hypoxemia during care, such as position changes. Additionally, the decision to initiate ECMO was made because she was young, had a single organ lesion, and the respiratory abnormalities were considered reversible. As a result, social reintegration was achieved despite the patient's immunodeficiency. Initiating ECMO in immunodeficient patients carries a high risk of increased complication rates and remains a topic of debate. Further accumulation of cases is necessary.

## Conclusions

In this case, we successfully treated severe PCP in an immunodeficient patient using ECMO and achieved social reintegration. Typically, the use of ECMO for severe respiratory failure in immunocompromised patients is considered a poor prognostic factor. However, while considering the indications for ECMO, there are cases like this one where flexible consideration of ECMO initiation is warranted.

## References

[1] Jiang Y, Huang X, Zhou H (2025). Clinical characteristics and prognosis of patients with severe pneumonia with Pneumocystis jirovecii colonization: a multicenter, retrospective study. Chest.

[2] Schmidt JJ, Lueck C, Ziesing S (2018). Clinical course, treatment and outcome of Pneumocystis pneumonia in immunocompromised adults: a retrospective analysis over 17 years. Crit Care.

[3] Ghembaza A, Vautier M, Cacoub P, Pourcher V, Saadoun D (2020). Risk factors and prevention of Pneumocystis jirovecii pneumonia in patients with autoimmune and inflammatory diseases. Chest.

[4] Cawcutt K, Gallo De Moraes A, Lee SJ, Park JG, Schears GJ, Nemergut ME (2014). The use of ECMO in HIV/AIDS with Pneumocystis jirovecii pneumonia: a case report and review of the literature. ASAIO J.

[5] Rilinger J, Staudacher DL, Rieg S, Duerschmied D, Bode C, Wengenmayer T (2019). Extracorporeal membrane oxygenation in Pneumocystis jirovecii pneumonia: outcome in HIV and non-HIV patients. Crit Care.

[6] Wang Y, Zhou X, Saimi M, Huang X, Sun T, Fan G, Zhan Q (2021). Risk factors of mortality from Pneumocystis pneumonia in non-HIV patients: a meta-analysis. Front Public Health.

[7] Tonna JE, Abrams D, Brodie D, Greenwood JC, Rubio Mateo-Sidron JA, Usman A, Fan E (2021). Management of adult patients supported with venovenous extracorporeal membrane oxygenation (VV ECMO): guideline from the Extracorporeal Life Support Organization (ELSO). ASAIO J.

